# Presentation, treatment, and outcome of renovascular hypertension below 2 years of age

**DOI:** 10.1007/s00431-022-04550-4

**Published:** 2022-07-06

**Authors:** Eda Didem Kurt-Sukur, Eileen Brennan, Meryl Davis, Colin Forman, George Hamilton, Nicos Kessaris, Stephen D. Marks, Clare A. McLaren, Kishore Minhas, Premal A. Patel, Derek J. Roebuck, Jelena Stojanovic, Sam Stuart, Kjell Tullus

**Affiliations:** 1 Department of Pediatric Nephrology, Hacettepe University School of Medicine, Ankara, Turkey; 2 Nephrology Unit, Great Ormond Street Hospital for Children, London, WC1N 3JH UK; 3 Department of Vascular Surgery, Royal Free Hospital, London, UK; 4 Department of Transplantation, Guy’s Hospital, Guy’s and St Thomas’ NHS Foundation Trust, London, UK; 5 NIHR Great Ormond Street Hospital Biomedical Research Centre, University College London Great Ormond Street Institute of Child Health, London, UK; 6 Department of Medical Imaging, Perth Children’s Hospital, Nedlands, 6009 Australia; 7 Curtin Medical School, Curtin University, Bentley, 6102 Australia; 8 Department of Interventional Radiology, Great Ormond Street Hospital for Children, NHS Foundation Trust, London, UK; 9 Division of Paediatrics, Medical School, University of Western Australia, Crawley, 6009 Australia

**Keywords:** Angioplasty, Endovascular, Hypertension, Pediatric, Renovascular

## Abstract

Renovascular hypertension in most cases requires endovascular treatment and/or surgery. This is technically much more difficult in small children and there is very limited published knowledge in this age group. We here present treatment and outcome of young children with renovascular hypertension at our institution. Children below 2 years of age, with renovascular hypertension between January 1998 and March 2020 were retrospectively reviewed. Demographics and treatment modalities were noted. Primary outcome was blood pressure within a week after the procedures and at last available visit. Sixty-six angiographies were performed in 34 patients. Median age at time of first angiography was 1.03 (interquartile range (IQR) 0.4–1.4) years and systolic blood pressure at presentation 130 (IQR 130–150) mm Hg. Thirty-eight percent (13/34) of children were incidentally diagnosed and 18% (6/34) presented with heart failure. Twenty-six (76%) children had main renal artery stenosis and 17 (50%) mid-aortic syndrome. Seventeen (50%) children showed intrarenal, six (18%) mesenteric, and three (9%) cerebrovascular involvement. Twenty patients underwent 45 percutaneous transluminal angioplasty procedures and seven children surgeries. In 44% of the 16 patients who underwent only percutaneous transluminal angioplasty blood pressure was normalized, 38% had improvement on same or decreased treatment and 19% showed no improvement. Complications were seen in 7.5% (5/66) of angiographies. In four of the seven (57%) children who underwent surgery blood pressure was normalized, two had improved (29%) and one unchanged (14%) blood pressure.

*Conclusion*: In small children with renovascular hypertension below the age of 2 years, percutaneous transluminal angioplasty caused significant improvement in blood pressure with low complication profile. Surgery can be recommended where percutaneous transluminal angioplasty and medical treatments failed.

**Table:** 

**What is Known:** *• Renovascular hypertension is diagnosed in all age groups from a few weeks of life until adulthood.* *• Both angioplasty and surgery are significantly more difficult to perform in small children and the published information on short and long-term outcome in these children is very scarce.*	
**What is New:** *• Children below the age of two years can safely and successfully undergo selective renal angiography and also safely be treated with angioplasty.* *• We here present a large group of babies and infants where angioplasty and in some cases surgery effectively and safely improved their blood pressure.*

**What is Known:**

*• Renovascular hypertension is diagnosed in all age groups from a few weeks of life until adulthood.*

*• Both angioplasty and surgery are significantly more difficult to perform in small children and the published information on short and long-term outcome in these children is very scarce.*

**What is New:**

*• Children below the age of two years can safely and successfully undergo selective renal angiography and also safely be treated with angioplasty.*

*• We here present a large group of babies and infants where angioplasty and in some cases surgery effectively and safely improved their blood pressure.*

## Introduction

Children with secondary hypertension have in 5–10% been found to have a renovascular disease [1]. Medical treatment of renovascular hypertension is usually not enough to normalize the blood pressure and angioplasty and/or surgery is needed [2–5].

Interventional treatment with percutaneous transluminal angioplasty has over the last decades become commonly used in children with renovascular hypertension while surgery previously was the treatment of choice [6–8]. Both percutaneous transluminal angioplasty and surgery are however technically challenging in small children with small blood vessels.

In the literature, there are very few patients under the age of 2 years [4, 9, 10] and there are no published studies focusing on the outcome in small children. The aim of this study was therefore to retrospectively evaluate the presentation, treatment, and outcome of children younger than 2 years of age who underwent diagnostic angiography as investigation of arterial hypertension at our institution.

## Materials and methods

Children with hypertension and a suspicion of a renovascular cause aged 2 years and younger at the time of intervention, who underwent digital substraction angiography between January 1998 and March 2020, were included in the study. The criteria used to perform the angiography were very high blood pressure, secondary symptoms of blood pressure like cardiac or neurological symptoms, uncontrolled hypertension with two or more antihypertensive drugs, diagnosis of a syndrome with a high risk of vascular disease, signs of vasculitis, previous vascular insult, bruit heard over renal arteries, raised plasma renin, or moderate hypokalemia [1]. Children with normal angiography findings and stenosis of transplant renal arteries were excluded. Data on patient demographics, clinical presentation, laboratory and radiologic investigations, treatment, and outcome were collected from medical records.

For patients older than 1 year of age, hypertension was defined when blood pressure was measured above the 95th percentile for individualized age-sex-height provided by Lurbe et al. [11]. In children under 1 year of age, 95th percentile of blood pressure was obtained from the second Task Force report [12].

### Angiography and angioplasty

All children underwent diagnostic angiography before any interventional procedures. In some patients, magnetic resonance (MR) or computerized tomography (CT) angiography were used for further anatomic evaluation of the abdominal vasculature. When clinically indicated cerebral MR angiography (MRA) or CT angiography (CTA) was performed.

Angioplasties were performed by a pediatric interventional radiologist under general anesthesia. Arterial access (usually femoral but left axillary if appropriate) was obtained by ultrasound guided insertion of a valved sheath. For angioplasty, balloon size was selected according to the estimated normal renal artery size and to avoid thrombosis aspirin was used.

### Outcome

Clinical outcome was graded according to suggestions of Ellis et al.: (1) normalized blood pressure (< 95th percentile for age, sex, and height), no antihypertensive treatment; (2) improved blood pressure with same or reduced treatment; (3) unchanged blood pressure or increased antihypertensive treatment; (4) technical failure (unable to pass the catheter or dilate the vessel) [13].

Short-term outcome was defined as blood pressure within a week after the procedures and long-term at last available visit. The Schwartz formula was used to calculate estimated glomerular filtration rate (eGFR) [14].

### Statistical analyses

Data analyses were performed by using SPSS Version 21.0 (IBM Corporation, Armonk, NYC, USA). Samples were tested with Shapiro–Wilk test to determine normality of distributions. According to the results, nonparametric tests were preferred. Continuous variables were compared by Mann–Whitney *U* test and categorical variables by Chi-square or Fisher’s exact test as appropriate. A *p* value of < 0.05 was considered statistically significant.

## Results

Thirty-seven children below the age of 2 years who underwent diagnostic angiography with suspected renovascular hypertension were assessed for eligibility. Among them three patients with normal angiography findings were excluded and a total of 34 patients were included in the final analysis. Male/female ratio was 15:19.

At the time of the first angiography at the authors’ institution four had had previous angioplasties. Seven children had a history of previous procedure or surgery. Nine children had an underlying syndrome; there were two cases of neurofibromatosis type 1 (NF-1), one Hurler’s syndrome, one Treacher-Collins syndrome, one Williams-Beuren syndrome, two other patients with phacomatoses and two patients with undefined syndromes.

At presentation, eight patients had elevated serum creatinine and two of them had known underlying renal parenchymal conditions. Two children had single kidneys, one unilateral hypoplasia and the other unilateral nephrectomy. Demographic, clinical, and laboratory characteristics of patients are given in Table 1.

**Table 1:** Demographic, clinical, and laboratory characteristics at the time of presentation; median (IQR)

Age (years)	1.03 (0.4–1.4)
Weight (kg)	8.6 (6.1–10.7)
Systolic blood pressure (mm Hg)	130 (130–150)
eGFR (mL/min/1.73 m^2^)	121 (88.7–155)
Serum creatinine (µmol/L)	28 (23–35)
Urine albumin/creatinine (mg/mmol)	4.3 (3–50.5)
Number of antihypertensive medications	3 (2–4)
Number of angiographies	1.5 (1–2)

*eGFR* estimated glomerular filtration rate

The clinical presentations of the children at the time of first angiography at the authors’ institution are shown in Table 2. There were two newborns in the cohort, both presented with collapse and hypertension. Nine children had cardiac symptoms of whom six presented with heart failure.

**Table 2:** Presenting features of children at the time of first angiography

Asymptomatic	13
Heart failure	6
Murmur	3
Lethargy-vomiting, poor feeding, failure to thrive	4
Acute hypertensive encephalopathy	3
Other neurological manifestations (facial palsy, seizures)	2
Cerebrovascular accident	2
During investigations for a syndrome	1

Results of echocardiography were available in 29 patients, 18 had mild left ventricular hypertrophy, six had moderate-severe cardiac involvement while five were normal.

Renal ultrasonography was performed in all children and was found to be normal on both sides in 16 patients and in both children with solitary kidneys. Pathological ultrasound findings with decreased renal size and/or increased echogenicity were seen in the remaining children. Ultrasound sensitivity was calculated as 44%. Renal Doppler ultrasound was performed in 30 patients and was normal in eight patients and in the two patients with solitary kidneys. Doppler ultrasound sensitivity was 63%. Dimercaptosuccinic acid (DMSA) scintigraphy was performed in 27 patients, was bilaterally normal in eight patients and normal in one of the patients with solitary kidney. Computerized tomography angiography was performed in four patients and missed intrarenal involvement in one patient. Magnetic resonance angiography was performed in two patients, diagnosed mid-aortic syndrome but missed renal artery stenosis in both.

### Angiographies

In total, 34 patients underwent 66 angiographies (Table 1). Nine patients underwent only diagnostic angiographies without any therapeutic approaches; in seven of them, the lesions were not amenable to treatment. The renal artery stenosis was bilateral in 15 patients, unilateral in eleven. Distribution of anatomical patterns of stenoses is given in Table 3.

**Table 3:** Anatomical pattern of stenoses at first angiographic examination and angioplasty

Stenosis pattern	Angiography	Angioplasty
Bilateral renal artery stenosis	15	9
Unilateral renal artery stenosis	11	9
Mid-aortic syndrome	17	11
Intrarenal involvement	17	6
Mesenteric involvement	6	3
Cerebrovascular involvement	3	-

### Angioplasties

**Fig. 1:**
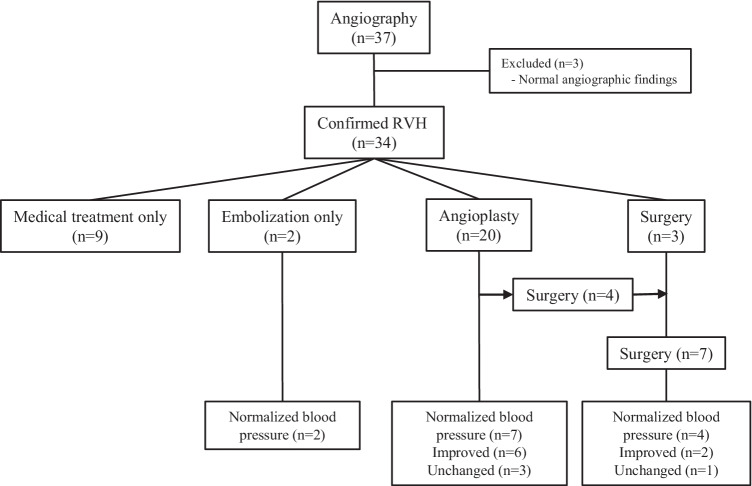
Last outcome of 34 patients according to treatment modalities. *RVH* renovascular hypertension

Twenty patients underwent 45 percutaneous transluminal angioplasties (Fig. 1). Eight patients underwent one angioplasty, eight patients had two angioplasties, one patient three, two patients five, and one patient had eight angioplasties. Table 3 depicts the stenoses patterns of patients who underwent angioplasty.

A cutting balloon was used three times. All three cutting balloon angioplasties were performed in patients diagnosed with phacomatoses (two with NF-1). Interventions on a previous vascular graft were performed in two angioplasty procedures. One child had one renal artery stented twice; the second time due to in-stent stenosis in the previously placed stent.

**Fig. 2:**
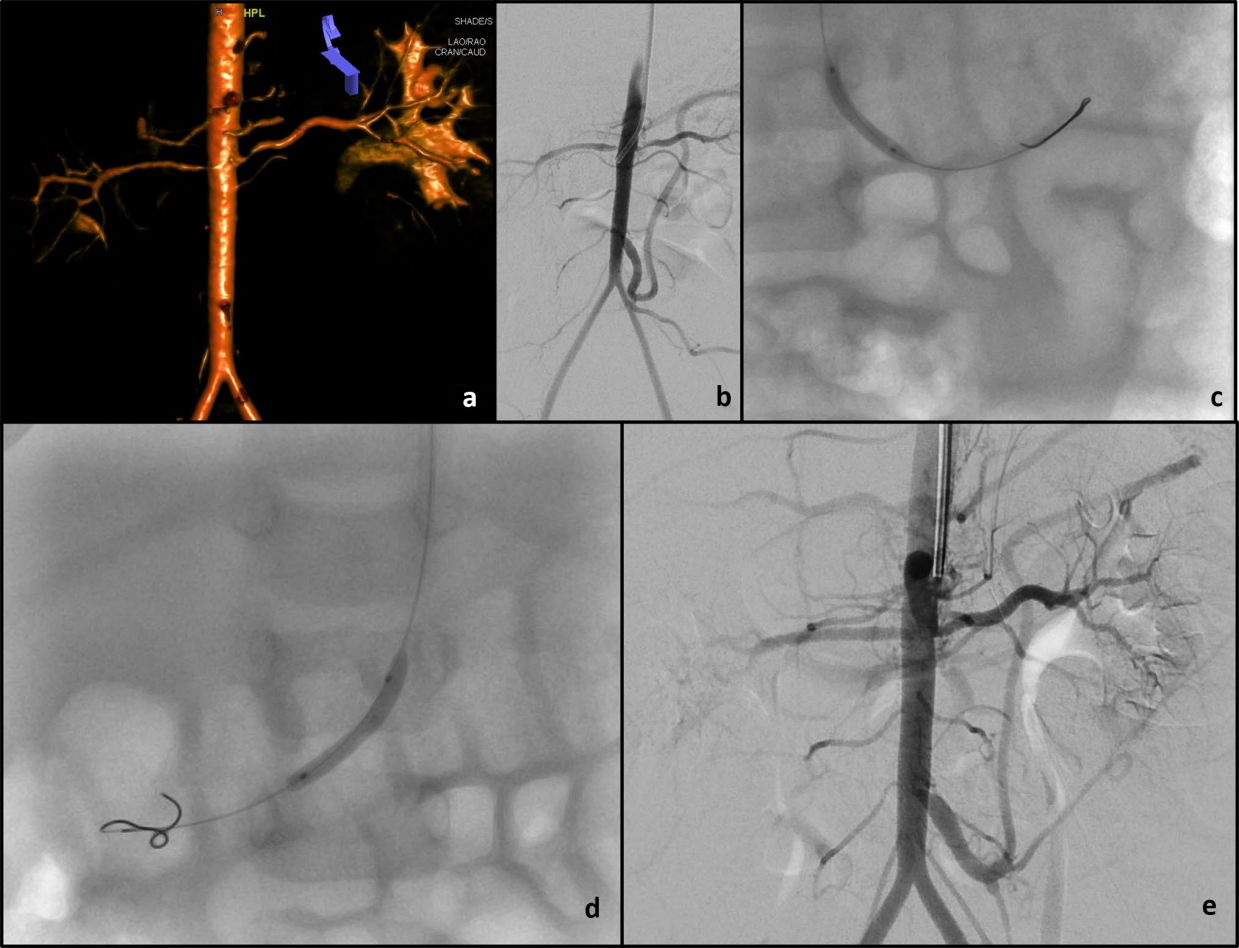
A 12-month-old infant presented with hypertension requiring four anti-hypertensives. Rotational angiogram (**a**) and digital subtraction aortogram (**b**) demonstrate a normal calibre abdominal aorta and bilateral renal artery osteal stenoses. Angioplasty of the left (**c**) and right (**d**) renal artery stenoses was performed with 2.0 mm and 2.25 mm balloons respectively using a left axillary artery approach. Completion digital subtraction angiogram (**e**) shows improved calibre of both renal artery ostia

**Fig. 3:**
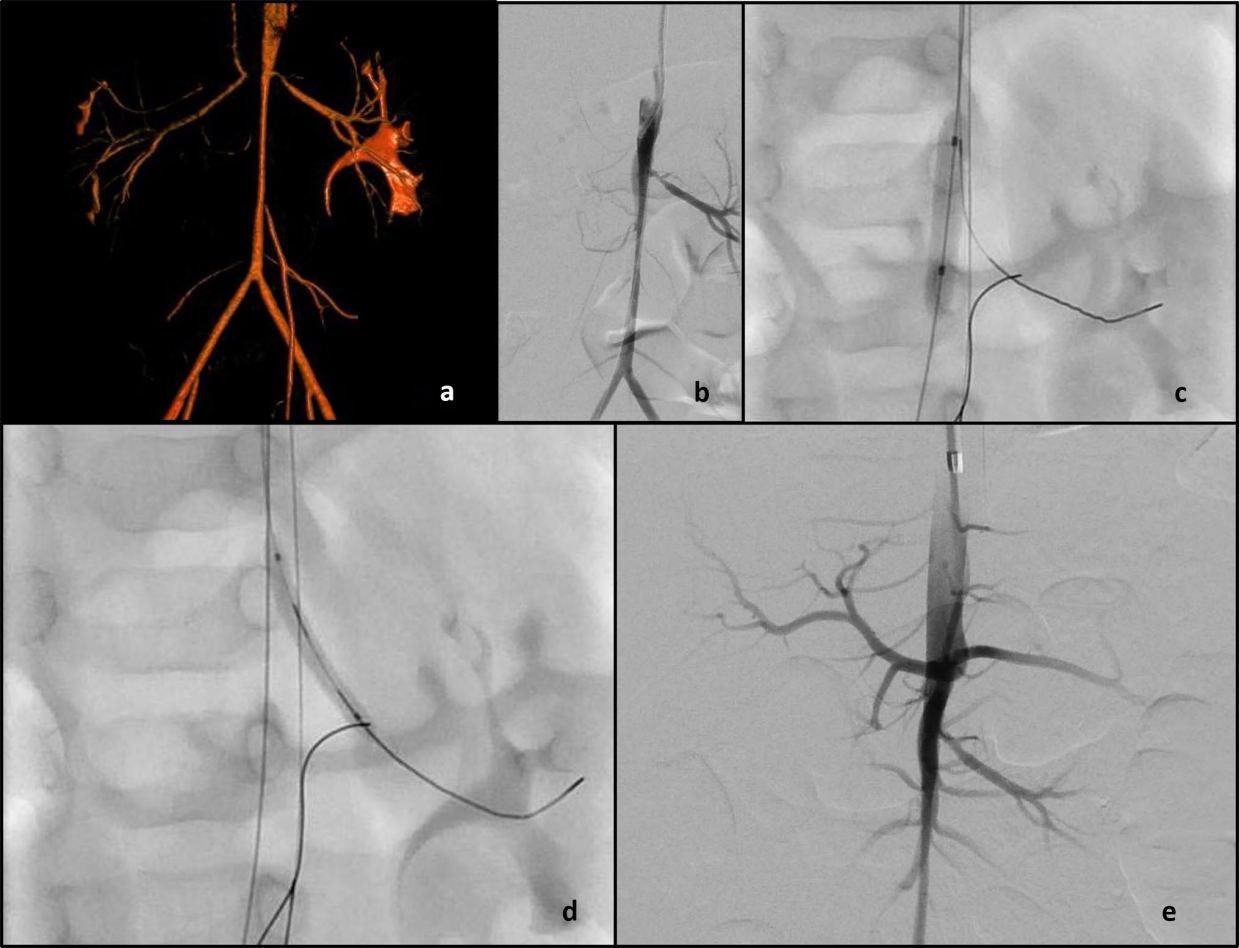
An 11-month-old infant, who was admitted to their local hospital with upper respiratory tract symptoms at the age of 6 months, was found to be hypertensive with a systolic blood pressure of 200 mmHg. Rotational angiogram (**a**) and digital subtraction aortogram (**b**) demonstrate middle aortic stenosis, stenosis of the left renal artery ostium and complete occlusion of the right renal artery origin with filling of the distal right renal artery via collaterals. Angioplasty of the juxtarenal abdominal aorta (**c**) with a 5 mm balloon, with safety wires in the left renal artery and superior mesenteric artery, was performed. Angioplasty of the left renal artery (**d**) with a 2.5 mm balloon was performed. Completion digital subtraction angiography (**e**) shows improved calibre of the juxtarenal abdominal aorta and left renal artery origin. Despite exhaustive efforts it was not possible to recanalize the occluded right renal artery (**b**, **e**)

The median time interval to second angioplasty after the first procedure was 5.5 (IQR 4–10) months. The indication for the second angioplasty was clinically resistant hypertension in all cases. An anatomical restenosis was found in three children and a significant residual stenosis in nine (Table 4). Mid-aortic syndrome was present in 4/5 of the patients who needed a third intervention. Three patients with multivessel involvement and one patient with unilateral renal artery stenosis in whom it was not possible to dilate the vessel required further surgical intervention. Figures 2 and 3 show examples of successful and failed angioplasties.

**Table 4:** Short-term outcome of the 20 children who had percutaneous transluminal angioplasty procedures

**Procedure**	**Total**	**BP normalized**	**BP improved on same or decreased treatment**	**BP unchanged or increased treatment**	**BP maintained > 95th percentile**	**Technical failure**
First PTA	20	3	9	6		2
Second PTA^a^	12		3	7		2
Third PTA^b^	5			5		
Fourth PTA^c^	3			3		
Fifth PTA	3		1	2		
Sixth PTA	1					1
Seventh PTA	1			1		
Eighth PTA	1			1		

*BP* blood pressure, *PTA* percutaneous transluminal angioplasty

^a^Indications for second PTA procedure: 3 restenosis, 9 remained significant

^b^Indications for third PTA procedure: 3 restenosis, 2 remained significant

^c^Indications for fourth PTA procedure: 2 restenosis, 1 remained significant

### Embolizations

Embolization (ethanol ablation) was used succesfully for three patients. In two patients with unilateral polar renal artery stenosis only embolization was performed. In the third patient, ethanol ablation was used after two angioplasties (one with a cutting-balloon) since the lesions were not amenable to angioplasty (Fig. 1).

### Complications

Among 66 angiographies (45 angioplasties), complications were seen in five procedures; arterial dissections treated with balloon angioplasty in three patients and thrombosis successfully treated with anticoagulation in two. No complications were observed beyond the 2nd angioplasty.

### Surgery

Seven children required surgical intervention. Three patients underwent unilateral nephrectomies. Four children had vascular surgery with one having surgical revascularization and unilateral nephrectomy (2.4 years old); one child underwent autotransplantation (6.5 years old); one patient had unilateral nephrectomy and an aortic graft (1.2 years old); and one had open thrombectomy (1 month old).

### Short-term outcome

Among the 20 patients who underwent angioplasty, after the first procedure, 15% (3/20) had normalized blood pressure, 45% (9/20) had blood pressure improvement on same or decreased treatment, 30% (6/20) showed no change in blood pressure or had increased treatment, and in 10% (2/20) technical failure was observed.

### Long-term outcome

The median time to last follow-up from the first procedure was 13 months (IQR 2.8–86) and from the last procedure 2.5 months (IQR 0.3–49). The median number of antihypertensive medications at last visit had decreased to 0.5 (IQR 0–3) from 3 (IQR 2–4) (*p* < 0.001).

At last visit, among the 16 patients who underwent only angioplasty, 44% (7/16) had normalized blood pressure, 37.5% (6/16) had blood pressure improvement on same or decreased treatment, and 18.5% (3/16) showed no change in blood pressure or had increased treatment. Of seven patients who underwent surgery, at last follow-up, four had normalized blood pressure, two had improved, and one had unchanged blood pressure values. All patients who underwent embolization normalized their blood pressure at last follow-up. Figure 1 shows the final outcome of the whole cohort.

The nine patients diagnosed with a syndrome showed a similar pattern of improvement in the blood pressure.

Table 4 shows the clinical outcome of angioplasty procedures. No further improvement of blood pressure was achieved after the second procedure. Neither the anatomical pattern of stenosis nor being younger than 1 year of age affected the last outcome significantly (*p* = 0.172 for age, *p* = 0.273 for diagnosis).

At last follow-up of the 25 patients who underwent any kind of intervention (embolization, percutaneous transluminal angioplasty, surgery), 52% had normalized blood pressure and 32% had blood pressure improvement on same or decreased treatment. Four patients had at final follow-up a blood pressure that was unchanged.

At last visit, median serum creatinine was 32 (IQR 26.5–53) µmol/L. There were three children (8%) with an eGFR of < 90 mL/min/1.73 m^2^, and median eGFR was 128 (IQR 91–146.5) mL/min/1.73 m^2^.

Death occured in four patients, none of which were procedure or hypertension related. One patient had neuroblastoma and died due to related complications. Another patient with Treacher-Collins syndrome died due to intracranial bleeding after an epileptic seizure. One patient died of multiorgan failure due to traumatic injury, and one patient with generalized arteriopathy and pulmonary hypertension died from heart failure.

## Discussion

In this study, we describe successful outcome of angioplasty and/or surgery or embolization in children with renovascular hypertension below the age of 2 years. Clinical success at last follow-up (normalized and improved blood pressure) was achieved in 84% (21/25) of the patients who underwent angioplasties and/or surgeries with an acceptable complication profile (5/66 angiographic interventions). There was no kidney loss or mortality related to the angiographic procedures.

An important number of the small children in our study presented with severe end-organ damage mainly in the heart with several having significant heart failure 18% and left ventricular hypertrophy in 83%. The indication for treatment was therefore strong. The literature on percutaneous transluminal angioplasty in small children is limited, with only some case reports and very few patients in some retrospective series [2, 9, 10, 15–17].

The success rate of percutaneous transluminal angioplasty in older children varies between 50 and 100% [5, 18–20]. In our study, blood pressure was normalized at last follow-up in 44% of the children after percutaneous transluminal angioplasty. The patients with unchanged blood pressure values had either multivessel involvement or complex vascular stenoses.

As seen also in older children, it is not uncommon that the percutaneous transluminal angioplasty needs to be repeated either due to restenosis or significant remaining stenosis after the preceding procedure [4, 5]. In the literature, recurrence of stenoses after the first percutaneous transluminal angioplasty ranged between 17 and 40% [4, 20, 21]. In this study, 12 patients underwent a second angioplasty with restenosis as the indication in 25%. Only eight patients needed only one angioplastic procedure. We found no further improvement of blood pressure after the second percutaneous transluminal angioplasty but another indication for further percutaneous transluminal angioplasty was to keep the vessel open until the child had grown to a size where surgery was possible. Surgical interventions in pediatric renovascular hypertension have also been shown to have high success rates. There is however in very young children and in children with mid-aortic syndrome a high risk both during surgery and of reoperation as the children grow out of their grafts [22].

There is no consensus regarding which diagnostic modalities to use in children with suspected renovascular hypertension. In our present study, ultrasound with Doppler had a low sensitivity of only 63%, which is similar to a previous study in children of all ages [23]. CTA and MRA are known to have limited success in small vessels and small children [1, 24]. For CTA and MRA, Trautman et al. found in older children with renal artery stenosis a sensitivity as of 88% for CTA and 80% for MRA, respectively [23]. In the present study, one out of four patients were missed with CTA. Angiography thus remains the gold standard for diagnosis of renovascular hypertension and also gives the additional advantage of possible treatment during the same procedure [23, 24].

End-organ damage is an important effect of systemic hypertension. In previous studies among older children, left ventricular hypertrophy was found in approximately 66% of patients [4, 5]. In this cohort, we found that 83% of the patients had some degree of cardiac involvement and six presented with heart failure.

The clinical success rate was also very good in our patients with a phacomatosis. This is a group who has been regarded as more difficult to treat than other children. We therefore suggest that also in young NF-1 patients percutaneous transluminal angioplasty can be a good option.

Complication rates of percutaneous transluminal angioplasty in children range between 0 and 43% [3, 9]. We found in this study a complication rate of 7.5%, all of which were successfully treated via angiographic approaches. None of the complications required surgical interventions. We have among 78 patients with a wider age-range (0.5–17 years) previously reported a complication rate of 11.4% [4]. We believe that technological improvements and increased institutional expertise has contributed to this positive result.

Limitations to our study are its retrospective design and relatively short follow-up period, and also the long time period of the study. Prospective studies with longer follow-ups preferrably into adulthood are required to be able to define the genuine rates of normalized blood pressure in pediatric patients.

In conclusion, angioplasty in infants below 2 years of age with renovascular hypertension, can be a safe and reliable treatment in experienced hands. However, its success rates decrease with multi-vessel and/or intrarenal involvement. Surgery can be recommended where percutaneous transluminal angioplasty and medical treatment are not effective to control blood pressure and preserve renal function.

## Data Availability

Excel data sheets can be made available upon request.

## Abbreviations

CT: Computerized tomography
CTA: Computerized tomography angiography
DMSA: Dimercaptosuccinic acid
eGFR: Estimated glomerular filtration rate
MR: Magnetic resonance
MRA: Magnetic resonance angiography
NF-1: Neurofibromatosis type 1
PTA: Percutaneous transluminal angioplasty
RVH: Renovascular hypertension
